# Rochester Academy of Medicine

**Published:** 1906-07

**Authors:** Henry T. Williams

**Affiliations:** Secretary


### Rochester Academy of Medicine

Reported By HENRY T. WILLIAMS, M. D., Secretary.

At a special meeting of the Rochester Academy of Medicine, held at Rochester, N. Y., June 1st, 1906, the following memorial was unanimously adopted:

IN MEMORIAM—LOUIS A. WEIGEL

By the untmely death of Dr. Louis A. Weigel the Rochester Academy of Medicine loses its president and one of its charter members. Each fellow of the academy has lost a personal friend and a valued consultant.

Though but 52 years old, Dr. Weigel had achieved a degree of professional success and honor accorded to very few. He was a specialist in the best sense of the word, for he had become such after years of general work and special preparation, so that as a diagnostician his outlook was never limited to the field of his specialty.

His reputation extended far beyond Rochester, for he had great mechanical skill as well as surgical knowledge and devised apparatus which is now used by orthopedic surgeons all over the world. The appreciation of his merit by the profession in general and by his fellow specialists has been shown by his election to the presidency of the American Orthopedic Society, by his appointment as professor of orthopedic surgery in Niagara University, by his position as consultant at the New York Hospital for Crippled Children and at Craig Colony.

Dr. Weigel lived his whole life in Rochester except for the time spent in the Medical Department of the University of Maryland. He accomplished much, not only because of special aptitude, but because he was a tremendous worker. Besides attending to his private practice he has for many years cared for those needing his special skill in the wards of the Rochester City Hospital and of St. Mary's Hospital and in the Free Out-Patient Department of the City Hospital. His hospital work was always done with the same care and interest as was his renumerative work, and much that he did outside the hospital brought little or no pecuniary reward.

As a member of the medical societies of Rochester, Dr. Weigel was of the greatest value to others in pointing out important preventive measures and diagnostic signs, and he was always ready to take his full share in the work at society meetings.

Dr. Weigel was a pioneer in the use of the Roentgen ray as an aid in diagnosis and as a therapeutic agent, and his unfortunate death may have been due to the prolonged irritation resulting from his continued peculiar professional work. He excelled not only because of his technical skill in photography, acquired years before, but largely on account of his artistic temperament.

The fortitude he displayed in enduring the keenest of anguish, and the moral courage he showed in facing the inevitable, brought out the high character of the man. His last months were brightened by the joy he found through the manifestation of the deepest human sympathy and the appreciation of his professional brethren.

It is with a profound sense of sorrow that we meet to do honor to his memory, and we extend to his family our sincere sympathy in their bereavement.

